# Associations between body mass index across adult life and hip shapes at age 60 to 64: Evidence from the 1946 British birth cohort

**DOI:** 10.1016/j.bone.2017.08.017

**Published:** 2017-12

**Authors:** Stella G. Muthuri, Fiona R. Saunders, Rebecca J. Hardy, Anastasia V. Pavlova, Kathryn R. Martin, Jennifer S. Gregory, Rebecca J. Barr, Judith E. Adams, Diana Kuh, Richard M. Aspden, Rachel Cooper

**Affiliations:** a MRC Unit for Lifelong Health and Ageing at UCL, 33 Bedford Place, London WC1B 5JU, UK; b Aberdeen Centre for Arthritis and Musculoskeletal Health, School of Medicine, Medical Sciences and Nutrition, University of Aberdeen, Aberdeen AB25 2ZD, UK.; c Medicines Monitoring Unit (MEMO), Division of Molecular & Clinical Medicine, School of Medicine, University of Dundee, Mailbox 2 Level 7, Ninewells Hospital & Medical School, Dundee DD1 9SY, UK; d Manchester Academic Health Science Centre and Radiology, Central Manchester University Hospitals NHS Foundation Trust and University of Manchester, Manchester Royal Infirmary, Oxford Road, Manchester M13 9WL, UK

**Keywords:** Life course epidemiology, Body mass index, Hip shape, Statistical shape modelling

## Abstract

**Objective:**

To examine the associations of body mass index (BMI) across adulthood with hip shapes at age 60–64 years.

**Methods:**

Up to 1633 men and women from the MRC National Survey of Health and Development with repeat measures of BMI across adulthood and posterior-anterior dual-energy X-ray absorptiometry bone mineral density images of the proximal femur recorded at age 60–64 were included in analyses. Statistical shape modelling was applied to quantify independent variations in hip mode (HM), of which the first 6 were examined in relation to: i) BMI at each age of assessment; ii) BMI gain during different phases of adulthood; iii) age first overweight.

**Results:**

Higher BMI at all ages (i.e. 15 to 60–64) and greater gains in BMI were associated with higher HM2 scores in both sexes (with positive HM2 values representing a shorter femoral neck and a wider and flatter femoral head). Similarly, younger age first overweight was associated with higher HM2 scores but only in men once current BMI was accounted for.

In men, higher BMI at all ages was also associated with lower HM4 scores (with negative HM4 values representing a flatter femoral head, a wider neck and smaller neck shaft angle) but no associations with BMI gain or prolonged exposure to high BMI were found. Less consistent evidence of associations was found between BMI and the other four HMs.

**Conclusion:**

These results suggest that BMI across adulthood may be associated with specific variations in hip shapes in early old age.

## Highlights

• BMI across adulthood was associated with variations in hip shape at age 60–64.
• BMI gain and prolonged exposure to overweight were also linked to hip shape.
• High BMI across adulthood may be associated with non-optimal shape of the hip joint.

## 1 Introduction

Obesity is a major global public health concern because of its negative impact on multiple disease processes and body systems. As the global prevalence of obesity continues to increase [1], [2], [3], disorders of the musculoskeletal system are expected to become more common especially in older populations at highest risk of these conditions [4], as excessive body weight may cause deleterious alterations to the structure of the bones and joints [5].

The relationship between obesity and chronic musculoskeletal conditions in adulthood has been widely studied. Epidemiological evidence has demonstrated that obesity is an important risk factor for development of knee [6], and hand osteoarthritis (OA) [7], with less consistent findings for hip OA [8], [9]. Obesity is also associated with increased risk of ankle and upper leg fractures [10], [11], whereas low BMI has been associated with increased risk of hip fracture in women [10], [12]. However, many of the studies examining associations of BMI with musculoskeletal outcomes have relied on the assessment of BMI at a single time-point. An exception are prior findings from the MRC National Survey of Health and Development (NSHD) that have shown associations of prolonged exposure to high BMI with increased risk of knee OA at age 53 [13], and decreased risk of low hip bone mineral density (BMD) [14].

The gross morphology of the proximal femur is a recognised factor in both hip OA and femoral neck fracture. Hip morphometry has traditionally used geometrical measures [15], [16], but recent studies using statistical shape modelling (SSM) have shown more subtle and coordinated changes in shape that are associated with fracture risk [17], [18], [19]. In addition, femoro-acetabular impingement, in which there is an incongruence between the shapes of the femoral head and the acetabulum, is increasingly recognised as a risk factor for hip OA [20], [21], and SSM has been able to provide quantitative measures of joint shape associated with risk of its incidence and progression [22], [23], [24], [25].

The relationships of BMI and joint shape with musculoskeletal diseases led us to hypothesise that BMI across adulthood may be associated with hip shape with important implications for bone and joint disorders in older adults. It is plausible that excess body weight may influence non-optimal shape of the hip joint via mechanical, inflammatory or other metabolic pathways. However, it is unclear whether associations exist between BMI across adulthood and hip shape in later life. It is also not clear whether greater gains in BMI during certain stages of adulthood or cumulative exposure to high BMI are particularly important. This information may provide insight about the most effective opportunities for intervention.

We utilised data from the NSHD to examine the relationships between BMI across adulthood and hip shapes at age 60–64. We aimed to investigate the associations of hip shapes at age 60–64 with: (i) BMI from age 15; (ii) BMI gain during different periods of adulthood, and (iii) length of exposure to overweight during adult life.

## 2 Methods

### 2.1 Study sample

The NSHD is a socially stratified sample of 5362 single, legitimate births that occurred in England, Wales and Scotland in one week of March 1946 and participants have been prospectively followed regularly ever since [26], [27]. Between 2006 and 2010 (at 60–64 years), eligible participants known to be alive and living in England, Wales and Scotland were invited for an assessment at one of six clinical research facilities (CRF) or to be visited at home by a research nurse. Of the 2856 invited, 2229 were assessed of whom 1690 attended a CRF. Approval for the study was obtained from the Central Manchester Research Ethics Committee (07/H1008/245) and the Scottish A Research Ethics Committee (08/MRE00/12) and written informed consent was obtained from each participant.

### 2.2 Radiological assessment

During the CRF visit, dual-energy X-ray absorptiometry (DXA) hip scans were acquired for BMD using a QDR 4500 Discovery scanner (Hologic Inc., Bedford, MA) by trained technicians following standard written and video protocols [14]. A single scan of the left hip was performed (unless contra-indicated by a prosthesis in the left hip, in which case the right hip was scanned (*n* = 63)). Individuals were scanned in the supine position with feet rotated internally by 15° and strapped to a foot brace placed centrally between the feet to ensure anteverted femoral was parallel to the table. All scans were assessed for image quality and quantitative analysis by JEA's laboratory.

For quality assurance, the Hologic Spine Phantom provided by the scanner manufacturer was scanned daily prior to participant scanning and in accordance with manufacturer's protocols, and the results were sent to the coordinating centre once a month for scrutiny [14]. Cross-calibration was achieved between centres using the European Spine Phantom [28].

### 2.3 Statistical shape modelling

Of the 1636 participants who had a hip DXA scan at age 60–64, 3 images were excluded after a review of all images and a consensus meeting of three investigators (RJB, JSG & FRS), due to extreme internal rotation of the joint shown by foreshortening of the femoral neck, leaving 1633 images to build the hip statistical shape modelling. SSM of these images has been described in detail previously [29]. Briefly, an SSM template consisting of 68 points was utilised. Procrustes transformation rotated and scaled the images before principal component analysis was applied to generate the independent orthogonal modes of variation. Each mode describes in descending order the percentage of variance standardised to a mean of 0 and standard deviation (SD) of 1. The variance was plotted against each mode in a scree plot and from this six modes of variance were selected for study using a cut-off of 5% variance; these six modes together explained 69% of overall shape variance.

### 2.4 Measurement of BMI and overweight history

Weight and height were measured by nurses using standardised protocols at ages 15, 36, 43, 53 and 60–64 years and self-reported at ages 20 and 26. BMI (weight (kg) / height (m^2^)) was then calculated at each age. BMI measures from age 20 were used to derive a variable with 6 categories indicating the age an individual first became overweight (i.e. BMI ≥ 25 kg/m^2^) as follows: 20 or 26; 36; 43; 53; 60–64; never overweight.

### 2.5 Statistical analysis

Linear regression models were used to investigate associations of BMI at each age (from 15 to 60–64) with each hip mode (HM) (aim 1). Tests of deviation from linearity were performed by including quadratic terms and, where evidence of this was found, tests for linear trend across sex-specific fifths were undertaken. At each age the maximum number of participants with valid data at that age were included in the model.

To investigate whether BMI gain during different periods of adulthood was differentially associated with each HM (aim 2), we first calculated change in BMI in early (20 to 36 years), mid (36 to 53 years) and later adulthood (53 to 60–64 years) conditional on earlier BMI by regressing each BMI measure on the earlier measure(s) for each sex and calculating the residuals. The residuals were standardised (mean = 0 and SD = 1) to allow comparability between the different periods [13]. Linear regression models that included the standardised residuals for all three intervals of BMI change and each HM were then run using the sample with complete data on BMI change and HMs (*n* = 1190). Wald tests were used to formally compare the coefficients.

To examine whether greater length of exposure to overweight during adult life was associated with each HM (aim 3), linear regression models were used to test the associations of the variable indicating age first overweight with each HM, using never overweight as the reference. These models were run unadjusted and also with adjustment for current BMI using a sample with complete data on overweight history and HMs (*n* = 1354).

All analyses were carried out separately for men and women, with sex interactions formally assessed using likelihood ratio tests (LRT). STATA v14.1 was used.

## 3 Results

The characteristics of the study sample are shown in Table 1. Mean BMI increased with increasing age and there were sex differences in BMI until age 53.

**Table 1:** Characteristics of participants from the MRC National Survey of Health and Development with data on hip shape at age 60–64, stratified by sex.

	Males		Females		*p*-value⁎
	N		N		
Sex, n (%)	779	779 (47.7)	854	854 (52.3)	
Age at nurse visit (years); mean (SD)	779	63.2 (1.15)	854	63.3 (1.07)	0.2
Mean (SD) BMI (kg/m^2^) at age (y);					
15	608	19.6 (2.36)	657	20.6 (2.74)	< 0.001
20	626	22.4 (2.32)	719	21.7 (2.76)	< 0.001
26	676	23.0 (2.68)	760	22.0 (2.93)	< 0.001
36	701	24.4 (2.87)	779	23.1 (3.41)	< 0.001
43	732	25.3 (3.08)	807	24.6 (4.13)	< 0.001
53	727	27.0 (3.65)	823	26.8 (5.0)	0.4
60–64	778	27.7 (3.94)	854	27.5 (5.17)	0.4
Age overweight (y); n (%)	653		701		< 0.001
Never overweight		109 (16.7)		179 (25.5)	
20 or 26		165 (25.3)		119 (17.0)	
36		136 (20.8)		79 (11.3)	
43		97 (14.9)		96 (13.7)	
53		103 (15.8)		163 (23.3)	
60–64		43 (6.5)		65 (9.3)	

Hip modes; mean (SD)					
HM1	779	0.22 (1.00)	854	− 0.20 (0.95)	< 0.001
HM2	779	0.20 (1.02)	854	− 0.18 (0.95)	< 0.001
HM3	779	− 0.25 (1.02)	854	0.23 (0.92)	< 0.001
HM4	779	0.22 (1.06)	854	− 0.20 (0.90)	< 0.001
HM5	779	0.02 (1.04)	854	− 0.02 (0.97)	0.4
HM6	779	0.20 (0.97)	854	− 0.18 (0.99)	< 0.001

⁎ comparison of sexes using student *t*-test or chi-square tests as appropriate.

**Fig. 1:**
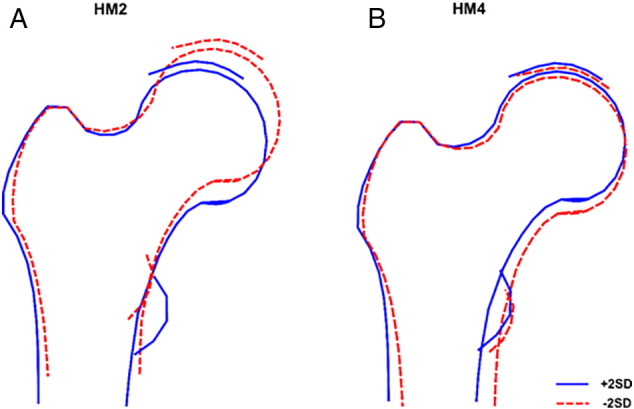
Line drawings of hip modes (HM) 2 and 4 showing ± 2 standard deviations (SD) from mean hip shape.

There were sex differences in all HMs except HM5 (Table 1). Men had positive mean scores for HM1, 2, 4 and 6 but negative mean scores for HM3, whereas women had negative mean scores for all modes except HM3 (Table 1). Descriptions and illustrations of key features of each HM are presented in Fig. 1, Table S1 and Fig. S1 [29].

### 3.1 HM 1

There were no associations between BMI and HM1, except at age 15 when higher BMI was weakly associated with higher HM1 scores in women (Table 2). Greater gains in BMI between ages 53 and 60–64 were associated with lower HM1 scores in men only (*p*-value for sex interaction = 0.05), with evidence that the effect in this age period was stronger than the effect between 36 and 53 (*p*-value from Wald test = 0.04) (Table 3). There was no evidence of association between age first overweight and HM1 scores in either sex (Table S2).

**Table 2:** Associations between BMI at ages 15 to 60–64 and hip modes (HM) 1 to 6 at 60–64 years in the MRC NSHD, by sex.

BMI per 1 kg/m^2^ at age:	Men	*P* value^a^	Women	*P* value^a^
	β (95%CI)		β (95%CI)	
HM 1				
15	0.014 (− 0.019, 0.048)	0.4	0.027 (0.001, 0.054)	0.04
20	0.010 (− 0.024, 0.045)	0.6	0.013 (− 0.012, 0.038)	0.3
26	0.016 (− 0.012, 0.044)	0.3	0.018 (− 0.005, 0.041)	0.1
36	− 0.005 (− 0.031, 0.020)	0.7	0.005 (− 0.015, 0.025)^c^	0.6
43	− 0.002 (− 0.025, 0.022)	0.9	0.002 (− 0.014, 0.018)^c^	0.8
53	0.003 (− 0.017, 0.023)	0.8	0.001 (− 0.012, 0.015)^c^	0.8
60–64	− 0.009 (− 0.027, 0.009)	0.3	0.001 (− 0.011, 0.013)	0.9

HM 2				
15	0.065 (0.031, 0.098)	< 0.001	0.075 (0.049, 0.101)	< 0.001
20	0.06 (0.025, 0.095)^c^	0.001	0.071 (0.046, 0.096)	< 0.001
26	0.071 (0.042, 0.099)	< 0.001	0.068 (0.045, 0.090)	< 0.001
36	0.065 (0.039, 0.091)	< 0.001	0.052 (0.033, 0.072)	< 0.001
43	0.054 (0.030, 0.077)	< 0.001	0.045 (0.030, 0.061)	< 0.001
53	0.050 (0.030, 0.07)	< 0.001	0.039 (0.027, 0.052)	< 0.001
60–64	0.048 (0.030, 0.066)	< 0.001	0.040 (0.028, 0.052)	< 0.001

HM 3				
15	− 0.001 (− 0.035, 0.033)^c^	0.9	− 0.022 (− 0.048, 0.003)	0.09
20^b^	− 0.032 (− 0.065, 0.001)	0.06	0.012 (− 0.013, 0.037)	0.3
26	− 0.0003 (− 0.028, 0.028)	> 0.9	0.015 (− 0.007, 0.038)	0.2
36^b^	− 0.016 (− 0.042, 0.009)^d^	0.2	0.018 (− 0.001, 0.037)	0.07
43^b^	− 0.017 (− 0.040, 0.007)^c^	0.2	0.016 (0.001, 0.032)	0.04
53^b^	− 0.011 (− 0.032, 0.009)	0.3	0.014 (0.001, 0.026)	0.03
60–64^b^	− 0.010 (− 0.028, 0.008)	0.3	0.015 (0.003, 0.027)	0.01

HM 4				
15^b^	− 0.094 (− 0.129, − 0.059)	< 0.001	− 0.006 (− 0.032, 0.019)	0.6
20^b^	− 0.052 (− 0.087, − 0.017)	0.004	− 0.0004 (− 0.024, 0.024)	> 0.9
26	− 0.036 (− 0.066, − 0.006)	0.02	− 0.008 (− 0.029, 0.014)	0.5
36^b^	− 0.043 (− 0.071, − 0.016)	0.002	0.005 (− 0.013, 0.023)	0.6
43^b^	− 0.039 (− 0.064, − 0.014)	0.002	0.002 (− 0.013, 0.017)	0.8
53^b^	− 0.035 (− 0.056, − 0.013)	0.001	0.005(− 0.007, 0.017)	0.4
60–64^b^	− 0.026 (− 0.045, − 0.007)	0.007	0.003 (− 0.009, 0.015)	0.6

HM 5				
15	0.031 (− 0.004, 0.067)	0.08	0.050 (0.024, 0.076)	< 0.001
20	0.02 (− 0.016, 0.056)	0.3	0.042 (0.017, 0.067)	0.001
26	0.014 (− 0.016, 0.043)	0.4	0.036 (0.013, 0.059)	0.002
36	0.019 (− 0.007, 0.046)	0.2	0.021 (0.001, 0.04)^c^	0.04
43	0.008 (− 0.017, 0.032)	0.5	0.011 (− 0.005, 0.028)	0.2
53	0.009 (− 0.011, 0.03)	0.4	0.007 (− 0.006, 0.021)	0.3
60–64	− 0.0003 (− 0.019, 0.018)^c^	> 0.9	0.012 (− 0.001, 0.024)	0.06

HM 6				
15	− 0.007 (− 0.039, 0.025)	0.7	− 0.015 (− 0.042, 0.013)	0.3
20	0.021 (− 0.012, 0.053)	0.2	− 0.001 (− 0.028, 0.025)	0.9
26	0.0002 (− 0.027, 0.027)	> 0.9	− 0.007 (− 0.031, 0.017)	0.6
36	0.020 (− 0.005, 0.044)	0.1	0.0003 (− 0.020, 0.021)	> 0.9
43	0.016 (− 0.007, 0.038)	0.2	− 0.003 (− 0.019, 0.014)	0.8
53	0.015 (− 0.004, 0.034)	0.1	0.005 (− 0.009, 0.019)	0.5
60–64	0.017 (− 0.001, 0.034)^c^	0.06	0.001 (− 0.012, 0.014)	0.8

Models are run on the maximum available N at each age (See Table 1).

a Wald's *p* value.

b sex-interaction *p* < 0.05.

c significant quadratic term but deviation from linearity was not confirmed when BMI was modelled as sex-specific fifths.

d non-linear relationship (LRT for quadratic term, *p* = 0.014) but weak overall LRT for BMI (*p* = 0.051).

**Table 3:** Associations between BMI gain during different periods of adulthood and hip modes 1 to 6 at age 60–64 in the MRC NSHD, by sex.

Interval of BMI change (age (y))	Men (*n* = 544)	*P* value†	Women (*n* = 646)	*P* value†
	β (95%CI)		β (95%CI)	
HM 1				
20 to 36	− 0.025 (− 0.115, 0.065)	0.6	− 0.002 (− 0.077, 0.072)	> 0.9
36 to 53	0.028 (− 0.064, 0.119)^a^	0.6	0.031 (− 0.043, 0.104)	0.4
53 to 60–64⁎	− 0.109 (− 0.200, − 0.018)	0.02	0.009 (− 0.067, 0.086)	0.8

HM 2				
20 to 36	0.149 (0.056, 0.242)^b^	0.002	0.104 (0.028, 0.18)	0.007
36 to 53	0.113 (0.017, 0.208)	0.020	0.108 (0.033, 0.183)	0.005
53 to 60–64	0.014 (− 0.08, 0.109)	0.8	0.083 (0.005, 0.16)	0.04

HM 3				
20 to 36	0.006 (− 0.08, 0.092)	0.9	0.097 (0.025, 0.169)	0.009
36 to 53	− 0.0005 (− 0.089, 0.088)	> 0.9	0.006 (− 0.066, 0.077)	0.9
53 to 60–64	− 0.011 (− 0.099, 0.077)	0.8	0.063 (− 0.012, 0.137)	0.1

HM 4				
20 to 36⁎	− 0.094 (− 0.189, 0.001)	0.05	0.035 (− 0.035, 0.106)	0.3
36 to 53	− 0.054 (− 0.151, 0.043)	0.3	0.002 (− 0.067, 0.072)	0.9
53 to 60–64	0.014 (− 0.082, 0.111)	0.8	0.012 (− 0.060, 0.085)	0.7

HM 5				
20 to 36	0.015 (− 0.08, 0.111)	0.7	0.015 (− 0.058, 0.089)	0.7
36 to 53	0.023 (− 0.075, 0.121)	0.6	0.007 (− 0.066, 0.08)	0.8
53 to 60–64	− 0.023 (− 0.12, 0.074)	0.6	0.029 (− 0.047, 0.104)	0.5

HM 6				
20 to 36	0.004 (− 0.08, 0.089)	0.9	− 0.001 (− 0.079, 0.077)	> 0.9
36 to 53	− 0.014 (− 0.1, 0.072)	0.7	0.004 (− 0.074, 0.081)	0.9
53 to 60–64	0.089 (0.003, 0.174)	0.04	− 0.008 (− 0.088, 0.072)	0.8

Notes: The coefficients from these models can be interpreted as the influence of change in BMI in the specified period above or below that expected given earlier BMI on HM scores.

Coef. > 0: greater gain in BMI (i.e. + 1 SD in BMI residuals) in the specified age interval associated with 1SD increase in the hip mode score.

Coef. < 0: greater gain in BMI (i.e. − 1 SD in BMI residuals) in the specified age interval associated with 1SD decrease in the hip mode score.

† From Wald test.

⁎ *P* value for sex interaction ≤ 0.05.

a Association between weight gain from 36 to 53 years and HM1 was larger than the association from 53 to 60–64years, (Wald test *p* value of the difference between the two coefficients = 0.04).

b Association between weight gain from 20 to 36 years and HM2 was larger than the association from 53 to 60–64years (Wald test *p* value of the difference between the two coefficients = 0.05).

### 3.2 HM 2

**Fig. 2:**
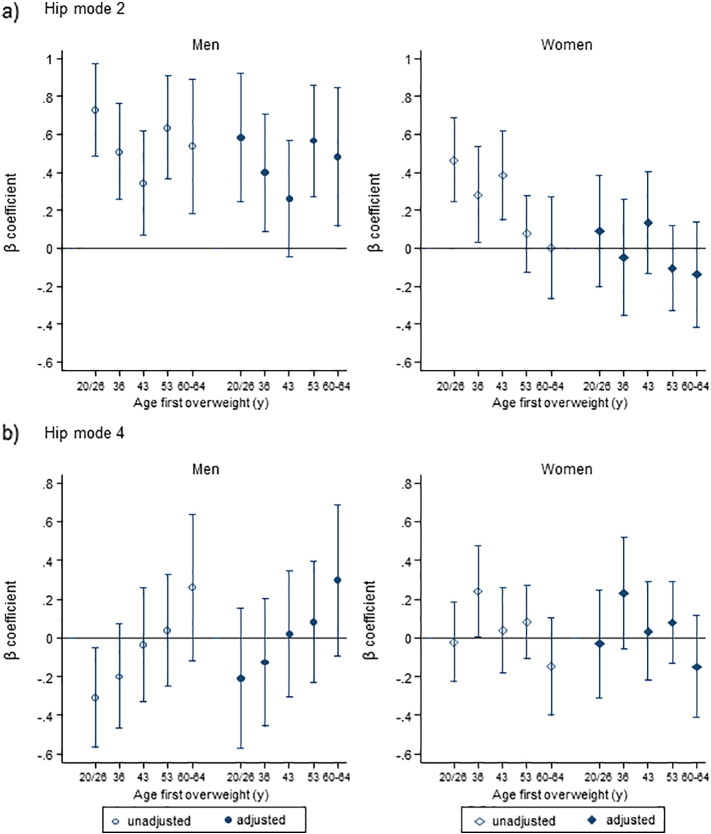
β coefficient and 95% CI per 1 SD increase in (a) HM2, (b) HM4, unadjusted (open markers) and adjusted for BMI at age 60–64 years (filled markers). Note: The point estimates show, from left to right, decreasing length of time since becoming overweight and the reference category was those who were never overweight or obese at all adult ages (from ages 20 onwards). Sample included 653 men and 701 women. Markers: circles (○) for men; diamonds (◊) for women. HM, hip mode.

At all ages, higher BMI was associated with higher HM2 scores in both sexes (*p*-values for sex interactions > 0.1) (Table 2). Greater gains in BMI across all three age periods were associated with higher HM2 scores in women and in the first two age periods in men (Table 3). Age first overweight was non-linearly associated with higher HM2 scores in both sexes with stronger associations observed in men (*p*-value for sex interaction = 0.003). Adjustment for current BMI attenuated associations in both sexes but non-linear relationships remained in men (Fig. 2(a), Table S2).

### 3.3 HM 3

Among men, there were no clear and consistent patterns of associations of BMI with HM3 (Table 2, Table 3 and S2). In contrast, higher BMI was associated with higher HM3 scores in women from age 20 with evidence that these associations were strengthening from age 43 (*p*-values for sex interactions < 0.01 except at ages 15 and 26) (Table 2). Greater BMI gain between ages 20 and 36 was associated with higher HM3 scores in women but no associations were observed with BMI gain during later periods (Table 3). No association was found between age overweight and HM3 in women (Table S2).

### 3.4 HM 4

Higher BMI at all adult ages was associated with lower HM4 scores in men (*p*-values for sex interactions < 0.05 except age 26) (Table 2). Greater BMI gain between ages 20 and 36 was weakly associated with lower HM4 scores in men but there were no associations with BMI gain at later ages (*p*-values from Wald tests > 0.1) (Table 3). Similarly, men who became overweight at younger ages had lower HM4 scores than those who were never overweight, and there was evidence of a linear trend (*p*-value for trend = 0.02) but this was attenuated after adjusting for current BMI (Fig. 2(b), Table S2). Among women, there were no clear and consistent patterns of associations of BMI with HM4 (Table 2, Table 3, S2 and Fig. 2(b)).

### 3.5 HM 5

Higher BMI up to and including age 36 was associated with higher HM5 scores in women but no associations were found between BMI at any age and HM5 in men (*p*-values for sex interactions > 0.2 at all ages) (Table 2). There was no evidence of association between BMI gain (Table 3) or age first overweight with HM5 in either sex (Table S2).

### 3.6 HM 6

Among men, there was some indication that higher BMI from age 20 was associated with higher HM6 scores, albeit non-significant (Table 2). Greater gains in BMI between ages 53 and 60–64, but not in other periods, were also weakly associated with higher HM6 scores in men; although there was little evidence to suggest differences in effect sizes in the three periods (*p*-values from Wald tests > 0.1) (Table 3). No linear association was found between age overweight and HM6 in men (Table S2).

In women, there were no clear and consistent patterns of associations of BMI with HM6 (Table 2, Table 3 and S2).

## 4 Discussion

### 4.1 Summary of main findings

To our knowledge, this is the first study to investigate the associations of BMI across adulthood and the impact of prolonged exposure to high BMI on hip shape in early old age. We found that higher BMI and greater BMI gain across adulthood was associated with higher HM2 scores in both sexes. Age at first becoming overweight was non-linearly associated with higher HM2 scores but this association was only retained in men once current BMI was accounted for. In women, there were also associations of higher BMI from age 43 and greater BMI gain in early adulthood with HM3; and for HM5 with higher BMI in early adulthood. In men, there were associations between higher BMI throughout adulthood and lower HM4 scores but no associations with BMI gain or prolonged exposure to high BMI. No clear evidence of associations were found between BMI and HM1 or HM6.

### 4.2 Explanation of findings

In our study, positive HM2 scores reflect a shorter femoral neck and wider and flatter femoral head (Fig. 1a, Table S1). These shapes are similar in appearance to those described for a cam or mixed femoro-acetabular impingement (FAI) which, in turn, may indicate a higher risk of OA. A cam FAI is characterised by thicker cortex in the lateral femoral neck resembling a ‘pistol-grip’ deformity [30], more common in men [20].

We found higher BMI at all ages, greater BMI gain until midlife, and younger age at first becoming overweight (independent of current BMI) to be associated with higher HM2 scores in men. In women, similar trends were evident except for age at becoming overweight which was explained by current BMI. That associations of age overweight with HM2 were fully attenuated after adjustment for current BMI in women but not in men may be due to stronger tracking of BMI across adulthood in women [13]. Alternatively, it could be explained by sex differences in body composition; although BMI is a widely used marker of adiposity in adults, higher BMI in earlier adulthood may be indicative of greater muscle mass in men. The consequence of this is that using a cut-point for overweight of ≥ 25 kg/m^2^ may have led to the misclassification of men with higher lean mass. This seems to be supported by findings from sensitivity analyses in which there was a non-linear association between age at first becoming obese (BMI ≥ 30 kg/m^2^) and higher HM2 scores in women only which attenuated after adjustment for current BMI (Table S3). In NSHD, detailed measures of body composition were only available at 60–64 years; associations observed between BMI and HM2 in men may be driven by lean, rather than fat mass, as further analyses showed a linear association between higher appendicular lean mass and higher HM2 scores in both sexes after adjusting for fat mass, whereas fat mass was associated with higher HM2 scores in women only (after adjusting for appendicular lean mass), (data not shown).

Our findings of linear associations across the full distribution of BMI also suggest that low BMI may have an effect on hip shape. Intriguingly, the shapes identified by scores at the negative end of HM2 (i.e. longer and thinner femoral necks with a greater neck-shaft angle) are similar to those previously found to confer a greater risk of hip fracture [17], [18]. In other studies, including measures of BMD and structure further improved the prediction of hip fracture [17], [31], but no corresponding correlation was found with low BMD [26], indicating that shape and BMD may be separately regulated.

In this study, negative values of HM4 represent flattening of the femoral head and a decrease in neck-shaft angle (Fig. 1b), shape changes that have previously been associated with OA [22]. We found sex differences in associations of HM4 with BMI at all ages and age first overweight, with much stronger, consistent evidence of associations in men. As for HM2, these differences may be due to sex differences in body composition with associations observed in men potentially related to variations in lean mass. That we also found sex differences with BMI gain in early adulthood lends this further support, as BMI gain in early adulthood in men may reflect the accrual of muscle mass.

Shapes described by positive scores for HM3 (Fig. S1 and Table S1) are similar to those describing a possible pincer FAI, with a greater extension to the acetabular rim. This is less common than a cam FAI and more common in middle-aged women [32]. In women, we found strong positive associations of HM3 with higher BMI in later midlife and greater gains in BMI in early adulthood but not with age first overweight. In addition, there was some evidence of associations for HM5 with higher BMI in early adulthood but not with BMI gain or age first overweight. These associations may be explained by BMI gain during childhood and/or puberty which tracked into adulthood. Previous research in this cohort has shown high relative weight in childhood was associated with high BMI in adulthood [33]. Similarly, other research in NSHD has shown that changes in BMI from childhood to adolescence were associated with knee OA at age 53 [13] and with hip BMD in women [14].

### 4.3 Methodological considerations

One key strength of this study is the use of a large nationally representative sample of older adults with repeat prospective measurements of BMI at regular intervals across adulthood which were assessed by research nurses at all but two ages.

Several important limitations must also be considered. Firstly, DXA hip images were captured at a single time-point. Therefore, we are unable to separate age-related morphologic changes from changes that may have occurred as a consequence of joint disorders such as hip OA. Secondly, although the SSM method has previously been used to identify individuals at risk of incidence and progression of hip OA [23], [24], [25], total hip replacement [22], or osteoporotic hip fracture [17], [18], [19], we lack data on these outcomes. This warrants future investigation; the hip shapes associated with obesity in this study have previously been associated with bone and joint disorders [17], [18], [19], [20], [21], [22], [23], [24], [25]. Thirdly, the amount of pelvic fat will affect the position of the joint between the beam source and the detectors and hence may introduce projection errors affecting the scaling. However, this will have been removed by the scaling process in the Procrustes analysis performed before PCA. In addition, although the feet are positioned routinely in internal rotation, we have no way of controlling for variation in the rotation of the proximal femur. Fourthly, our analyses were minimally adjusted and so findings may be explained by residual confounding by factors such as socioeconomic position, physical activity and BMD. Fourthly, in this cohort few individuals remained underweight (BMI < 20 kg/m^2^) across adulthood whereby we were unable to reliably determine the risk associated with prolonged exposure to underweight. In addition, the measure of overweight history used in these analyses assumed that once an individual became overweight they remained overweight. When this assumption was tested we found it to hold; 79% of those classified as first overweight between ages 20/26 and 53 were overweight at all subsequent assessments. Further sensitivity analyses excluding those participants who were not consistently overweight showed similar associations (data not shown) when compared with findings which included all participants (Table S2), also suggesting that this assumption was reasonably well met. Another limitation is that our analyses were restricted to the sample who attended a CRF as this is where DXA scans were undertaken. It is possible that this restriction may have introduced bias as participants who attended a CRF were less likely to be obese and more likely to be in better health than those who were visited at home [34]. Lastly, our study population comprised Caucasian men and women in early old age and although we stratified our analyses by sex [15], hip shapes are also likely to vary by age and ethnicity [35]. However, a benefit of this is that we can be confident that our findings are not explained by confounding by age or ethnicity and, as the sample has remained broadly representative of the population from which it was drawn [34], they are likely to be generalisable to the UK population born at a similar time.

## 5 Conclusion

Our results suggest that BMI across adulthood is associated with specific variations in hip shape at age 60–64. Future prospective studies, especially those with repeat measures of fat and lean mass across life and with data on outcome measures relating to the hip joint (e.g. hip OA, osteoporotic hip fracture) are required to confirm these findings and assess their clinical implications.

## Funding

The NSHD is funded by the UK Medical Research Council. SGM, RC, RJH and DK are supported by the UK Medical Research Council (Programme codes: MC_UU_12019/1, MC_UU_12019/2 and MC_UU_12019/4). This project was funded by the UK Medical Research Council (Grant MR/L010399/1) which supported SGM, AVP and FRS. The funders of the study had no role in study design, data collection, data analysis, data interpretation or writing of this manuscript.

## Author contributions

Conception and study design: DK, RH, SGM, JSG, RJB, RC, RMA. Acquisition of data: DK, RH, JEA. Derivation of hip shapes: FRS, AVP, JSG, RJB, RMA. Analysis of data: SGM. Interpretation of data: All authors. Drafting of the manuscript: SGM. Critical revision of manuscript and approval of final version to be published: All authors. Final approval of the version published: All authors.

SGM had full access to all the data in the study and takes full responsibility for the integrity and the accuracy of data analysis.

## Competing interests

All authors have no conflicts of interest to declare.

## Appendix A Supplementary data

Supplementary material Image 1
