# Supplementary material for: Associations between body mass index across adult life and hip shapes at age 60 to 64: Evidence from the 1946 British birth cohort
Source: Bone. 2017 Dec;105:115–21. doi: 10.1016/j.bone.2017.08.017 (PMC5658512; doi:10.1016/j.bone.2017.08.017)
Supplement: Supplementary file 1 — Supplementary material [file mmc1.docx]

**SUPPLEMENTARY MATERIAL**

Table S1: Characteristics of hip modes (HM) 1 to 6 in the MRC National Survey of Health and Development

| HM | Lower values | Higher values |
| --- | --- | --- |
| 1 | - compact femoral head - larger neck shaft angle | - wider femoral neck - smaller neck shaft angle - increased prevalence of osteophytes |
| 2 | - longer femoral neck, - loss of femoral head curvature - increased external rotation | - wider and larger greater and lesser trochanters - wider femoral head and neck - increased prevalence of osteophytes |
| 3 | - larger femoral head - loss of curvature around the femoral head and neck - wider femoral neck | - greater acetabular coverage of the femoral head - smaller neck shaft angle |
| 4 | - flatter femoral head - wider neck - smaller neck shaft angle | - increased inferior osteophytes - small increase in acetabular coverage |
| 5 | - More evident lesser trochanter | - slight flattening of the femoral head - increased prevalence of osteophytes |
| 6 | - changes to the femoral head curvature - altered joint rotation | - normal curvature of the femoral head - normal rotation |

Figure S1. Line drawings of hip modes (HM) showing ±2 standard deviations (SD) from mean hip shape. Percentage of variance explained by each mode is shown in brackets.


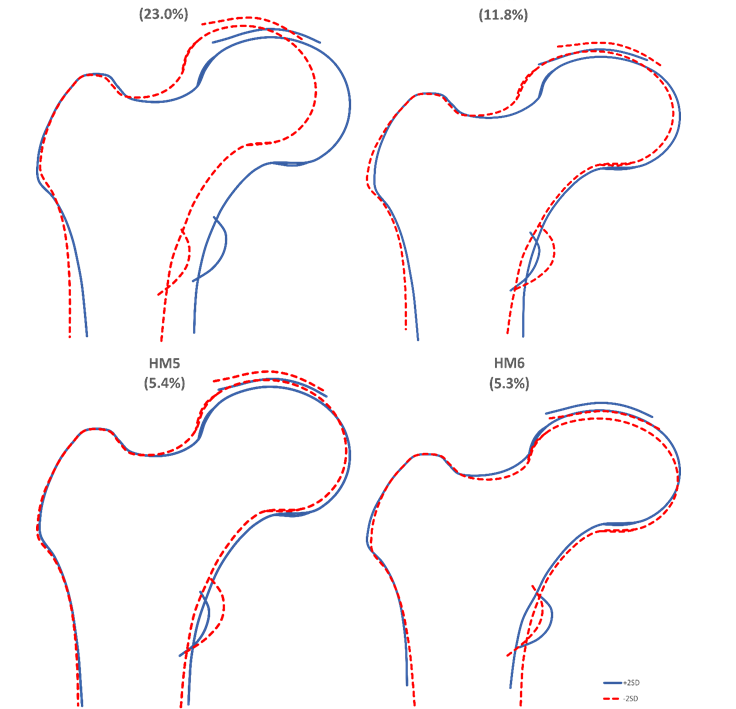


Table S2: Associations between age first overweight and hip modes 1 to 6 at age 60-64 in the MRC NSHD, by sex

|  | **Mode 1** | **Mode 2** | **Mode 3** | **Mode 4** | **Mode 5** | **Mode 6** |
| --- | --- | --- | --- | --- | --- | --- |
|  | β (95%CI) | β (95%CI) | β (95%CI) | β (95%CI) | β (95%CI) | β (95%CI) |
| ***Unadjusted*** |  |  |  |  |  |  |
| **Men** (n=653) |  |  |  |  |  |  |
| Never overweight^†^ | ref | ref | ref | ref | ref | ref |
| Overweight 20 or 26y | 0.094 (-0.15 , 0.337) | 0.728 (0.485 , 0.970)* | -0.085 (-0.318 , 0.149) | -0.306 (-0.565 , -0.047)* | 0.019 (-0.234 , 0.271) | 0.156 (-0.074 , 0.385) |
| Overweight 36y | -0.061 (-0.315 , 0.192) | 0.510 (0.257 , 0.762)* | 0.026 (-0.217 , 0.269) | -0.198 (-0.468 , 0.071) | 0.092 (-0.171 , 0.354) | 0.268 (0.029 , 0.507)* |
| Overweight 43 y | -0.017 (-0.292 , 0.258) | 0.342 (0.068 , 0.616)* | -0.141 (-0.405 , 0.123) | -0.033 (-0.326 , 0.259) | -0.014 (-0.299 , 0.271) | 0.126 (-0.133 , 0.385) |
| Overweight 53y | -0.023 (-0.294 , 0.248) | 0.637 (0.367 , 0.907)* | -0.251 (-0.511 , 0.008) | 0.038 (-0.250 , 0.326) | -0.186 (-0.467 , 0.095) | 0.075 (-0.181 , 0.33) |
| Overweight 60-64y | 0.125 (-0.23 , 0.48) | 0.538 (0.184 , 0.892)* | -0.233 (-0.574 , 0.107) | 0.262 (-0.116 , 0.639) | 0.174 (-0.193 , 0.542) | -0.106 (-0.441 , 0.229) |
| P for trend (except‡) | .87 | <.001‡ | .04 | .02 | .61 | .63 |
|  |  |  |  |  |  |  |
| **Women** (n= 701) |  |  |  |  |  |  |
| Never overweight^†^ | ref | ref | ref | ref | ref | ref |
| Overweight 20 or 26y | 0.168 (-0.055 , 0.391) | 0.465 (0.245 , 0.685)* | 0.175 (-0.036 , 0.387) | -0.020 (-0.227 , 0.186) | 0.299 (0.079 , 0.518)* | -0.071 (-0.303 , 0.162) |
| Overweight 36y | 0.090 (-0.164 , 0.345) | 0.282 (0.030 , 0.533)* | 0.247 (0.006 , 0.488)* | 0.241 (0.005 , 0.477)* | 0.175 (-0.076 , 0.425) | 0.132 (-0.133 , 0.398) |
| Overweight 43 y | 0.175 (-0.064 , 0.413) | 0.384 (0.149 , 0.620)* | 0.137 (-0.089 , 0.363) | 0.040 (-0.181 , 0.261) | 0.099 (-0.136 , 0.334) | -0.00002 (-0.249 , 0.249) |
| Overweight 53y | 0.057 (-0.147 , 0.261) | 0.077 (-0.124 , 0.279) | 0.13 (-0.064 , 0.323) | 0.085 (-0.104 , 0.274) | 0.063 (-0.138 , 0.264) | -0.09 (-0.303 , 0.123) |
| Overweight 60-64y | -0.186 (-0.458 , 0.087) | 0.004 (-0.266 , 0.274) | 0.198 (-0.061 , 0.457) | -0.145 (-0.398 , 0.108) | 0.141 (-0.127 , 0.41) | -0.081 (-0.366 , 0.204) |
| P for trend (except‡) | .51 | <.001‡ | .19 | .09‡ | .09‡ | .48 |
| P for sex interaction | .38 | .004 | .21 | .006 | .48 | .76 |
|  |  |  |  |  |  |  |
| ***Adjusted for current BMI*** |  |  |  |  |  |  |
| **Men** (n=653) |  |  |  |  |  |  |
| Never overweight^†^ | ref | ref | ref | ref | ref | ref |
| Overweight 20 or 26y | 0.337 (-0.002 , 0.676) | 0.583 (0.244 , 0.922)* | 0.106 (-0.22 , 0.431) | -0.210 (-0.571 , 0.152) | 0.017 (-0.336 , 0.369) | 0.050 (-0.27 , 0.371) |
| Overweight 36y | 0.124 (-0.186 , 0.434) | 0.400 (0.090 , 0.710)* | 0.171 (-0.127 , 0.469) | -0.125 (-0.456 , 0.206) | 0.09 (-0.233 , 0.413) | 0.188 (-0.105 , 0.481) |
| Overweight 43 y | 0.122 (-0.184 , 0.427) | 0.260 (-0.046 , 0.565) | -0.032 (-0.326 , 0.261) | 0.021 (-0.304 , 0.347) | -0.016 (-0.333 , 0.302) | 0.066 (-0.223 , 0.355) |
| Overweight 53y | 0.096 (-0.198 , 0.389) | 0.567 (0.273 , 0.860)* | -0.158 (-0.441 , 0.124) | 0.085 (-0.228 , 0.398) | -0.187 (-0.493 , 0.119) | 0.023 (-0.254 , 0.301) |
| Overweight 60-64y | 0.218 (-0.147 , 0.583) | 0.483 (0.118 , 0.848)* | -0.161 (-0.511 , 0.19) | 0.298 (-0.091 , 0.688) | 0.174 (-0.206 , 0.554) | -0.146 (-0.492 , 0.199) |
| P for trend (except‡) | .89 | .008‡ | .04 | .02 | .61 | .60 |
|  |  |  |  |  |  |  |
| **Women** (n=701) |  |  |  |  |  |  |
| Never overweight^†^ | ref | ref | ref | ref | ref | ref |
| Overweight 20 or 26y | 0.268 (-0.032 , 0.569) | 0.092 (-0.203 , 0.386) | 0.033 (-0.252 , 0.318) | -0.029 (-0.308 , 0.250) | 0.320 (0.023 , 0.616)* | -0.118 (-0.432 , 0.197) |
| Overweight 36y | 0.179 (-0.132 , 0.49) | -0.049 (-0.354 , 0.256) | 0.121 (-0.174 , 0.415) | 0.233 (-0.056 , 0.522) | 0.193 (-0.114 , 0.500) | 0.09 (-0.235 , 0.416) |
| Overweight 43 y | 0.241 (-0.032 , 0.515) | 0.135 (-0.133 , 0.403) | 0.042 (-0.218 , 0.301) | 0.035 (-0.220 , 0.289) | 0.113 (-0.157 , 0.383) | -0.032 (-0.318 , 0.255) |
| Overweight 53y | 0.106 (-0.121 , 0.332) | -0.105 (-0.327 , 0.117) | 0.060 (-0.155 , 0.275) | 0.081 (-0.130 , 0.291) | 0.073 (-0.150 , 0.296) | -0.113 (-0.35 , 0.124) |
| Overweight 60-64y | -0.148 (-0.431 , 0.135) | -0.138 (-0.416 , 0.140) | 0.144 (-0.125 , 0.412) | -0.148 (-0.411 , 0.115) | 0.149 (-0.130 , 0.429) | -0.099 (-0.396 , 0.197) |
| P for trend (except‡) | .44 | .37‡ | .37 | .10‡ | .26‡ | .43 |
| P for sex interaction | .29 | .003 | .22 | .006 | .48 | .72 |

*p≤.05; † BMI <25kg/m^2^; ‡ p value for test of heterogeneity across groups when there was evidence of a deviation from a linear trend

Table S3: Associations between age first obese (i.e. BMI≥30kg/m^2^) and hip modes 1 to 6 at age 60-64 in the MRC NSHD, by sex

|  | **Mode 1** | **Mode 2** | **Mode 3** | **Mode 4** | **Mode 5** | **Mode 6** |
| --- | --- | --- | --- | --- | --- | --- |
|  | β (95%CI) | β (95%CI) | β (95%CI) | β (95%CI) | β (95%CI) | β (95%CI) |
| ***Unadjusted*** |  |  |  |  |  |  |
| **Men** (n=594) |  |  |  |  |  |  |
| Never obese^‡^ | ref | ref | ref | ref | ref | ref |
| Obese 26 or 36y | 0.244 (-0.111 , 0.599) | 0.480 (0.108 , 0.852)* | -0.110 (-0.466 , 0.247) | -0.382 (-0.778 , 0.013) | 0.312 (-0.075 , 0.700) | -0.058 (-0.405 , 0.288) |
| Obese 43 y | -0.219 (-0.598 , 0.159) | 0.284 (-0.112 , 0.681) | -0.212 (-0.592 , 0.169) | -0.226 (-0.648 , 0.196) | -0.079 (-0.493 , 0.334) | 0.159 (-0.211 , 0.528) |
| Obese 53y | 0.066 (-0.194 , 0.325) | 0.351 (0.079 , 0.623)* | -0.167 (-0.428 , 0.094) | -0.306 (-0.595 , -0.016)* | 0.276 (-0.008 , 0.559) | 0.085 (-0.168 , 0.339) |
| Obese 60-64y | -0.181 (-0.444 , 0.082) | 0.305 (0.029 , 0.58)* | -0.004 (-0.268 , 0.261) | -0.06 (-0.354 , 0.233) | -0.164 (-0.451 , 0.124) | 0.142 (-0.115 , 0.398) |
| P for trend (except‡) | .18‡ | .17‡ | .38 | .11 | .04‡ | .20 |
|  |  |  |  |  |  |  |
| **Women** (n= 675) |  |  |  |  |  |  |
| Never obese^‡^ | ref | ref | ref | ref | ref | ref |
| Obese 26 or 36y | -0.230 (-0.586 , 0.126) | 0.660 (0.311 , 1.010)* | 0.355 (0.017 , 0.693)* | -0.073 (-0.403 , 0.257) | 0.213 (-0.136 , 0.561) | -0.103 (-0.470 , 0.264) |
| Obese 43 y | 0.133 (-0.154 , 0.419) | 0.329 (0.048 , 0.611)* | 0.165 (-0.107 , 0.437) | 0.028 (-0.238 , 0.293) | -0.028 (-0.309 , 0.252) | -0.091 (-0.387 , 0.204) |
| Obese 53y | 0.099 (-0.134 , 0.331) | 0.465 (0.237 , 0.694)* | -0.010 (-0.231 , 0.211) | 0.133 (-0.083 , 0.349) | 0.142 (-0.086 , 0.369) | 0.055 (-0.185 , 0.295) |
| Obese 60-64y | 0.122 (-0.138 , 0.381) | 0.348 (0.094 , 0.603)* | -0.042 (-0.288 , 0.204) | -0.009 (-0.25 , 0.231) | -0.071 (-0.325 , 0.182) | 0.048 (-0.220 , 0.315) |
| P for trend (except‡) | .48‡ | .009‡ | .99 | .52 | .33‡ | .73 |
|  |  |  |  |  |  |  |
| P for sex interaction | .07 | .93 | .17 | .12 | .90 | .87 |
|  |  |  |  |  |  |  |
|  |  |  |  |  |  |  |
| ***Adjusted for current BMI*** |  |  |  |  |  |  |
| **Men** (n=594) |  |  |  |  |  |  |
| Never obese^‡^ | ref | ref | ref | ref | ref | ref |
| Obese 26 or 36y | 0.494 (0.042 , 0.947)* | 0.0003 (-0.471 , 0.472) | -0.082 (-0.538 , 0.375) | -0.196 (-0.701 , 0.31) | 0.256 (-0.24 , 0.752) | -0.365 (-0.807 , 0.076) |
| Obese 43 y | -0.01 (-0.456 , 0.435) | -0.116 (-0.58 , 0.348) | -0.189 (-0.637 , 0.26) | -0.07 (-0.567 , 0.427) | -0.126 (-0.613 , 0.361) | -0.097 (-0.531 , 0.336) |
| Obese 53y | 0.247 (-0.083 , 0.577) | 0.004 (-0.34 , 0.347) | -0.147 (-0.479 , 0.185) | -0.171 (-0.539 , 0.198) | 0.235 (-0.126 , 0.596) | -0.137 (-0.458 , 0.184) |
| Obese 60-64y | -0.018 (-0.339 , 0.303) | -0.008 (-0.342 , 0.326) | 0.014 (-0.309 , 0.338) | 0.061 (-0.297 , 0.42) | -0.2 (-0.551 , 0.151) | -0.059 (-0.371 , 0.254) |
| P for trend (except‡) | .06‡ | .96‡ | .87 | .85 | .09‡ | .97 |
|  |  |  |  |  |  |  |
| **Women** (n= 675) |  |  |  |  |  |  |
| Never obese^‡^ | ref | ref | ref | ref | ref | ref |
| Obese 26 or 36y | -0.381 (-0.859 , 0.097) | 0.394 (-0.075 , 0.863) | 0.011 (-0.441 , 0.463) | -0.157 (-0.6 , 0.286) | -0.017 (-0.484 , 0.451) | -0.202 (-0.695 , 0.292) |
| Obese 43 y | 0.025 (-0.342 , 0.391) | 0.138 (-0.222 , 0.498) | -0.082 (-0.429 , 0.265) | -0.032 (-0.372 , 0.308) | -0.193 (-0.552 , 0.166) | -0.162 (-0.541 , 0.216) |
| Obese 53y | 0.011 (-0.286 , 0.309) | 0.31 (0.019 , 0.602)* | -0.21 (-0.492 , 0.071) | 0.084 (-0.191 , 0.36) | 0.008 (-0.283 , 0.299) | -0.002 (-0.309 , 0.305) |
| Obese 60-64y | 0.049 (-0.253 , 0.351) | 0.219 (-0.077 , 0.515) | -0.209 (-0.495 , 0.077) | -0.05 (-0.33 , 0.23) | -0.183 (-0.478 , 0.113) | 0 (-0.312 , 0.312) |
| P for trend (except‡) | .34‡ | .39‡ | .048 | .74 | .56‡ | .73 |
|  |  |  |  |  |  |  |
| P for sex interaction | .07 | .997 | .33 | .11 | .85 | .77 |

*p ≤.05; ‡ BMI <30kg/m^2^
